# Three single nucleotide polymorphisms of TNFAIP3 gene increase the risk of rheumatoid arthritis

**DOI:** 10.18632/oncotarget.15265

**Published:** 2017-02-10

**Authors:** Nan Shen, Yuan Ruan, Yajun Lu, Xuefeng Jiang, Huiqing Sun, Gongming Gao, Luming Nong, Kewei Ren

**Affiliations:** ^1^ Department of Clinical Pharmacy, The Affiliated Jiangyin Hospital of Southeast University Medical School, Jiangyin 214400, China; ^2^ Department of Minimally Invasive Spine Center, Renji Orthopedics Hospital, Shantou 515065, China; ^3^ Department of Orthopedics, Jiangyin No 3 People's Hospital, Jiangyin 214433, China; ^4^ Department of Orthopedics, The Affiliated Jiangyin Hospital of Southeast University Medical School, Jiangyin 214400, China; ^5^ Department of Orthopedics, The Affiliated Changzhou No 2 Hospital of Nanjing Medical University, Changzhou 213003, China

**Keywords:** TNFAIP3, single nucleotide polymorphism, rheumatoid arthritis, meta-analysis

## Abstract

Rheumatoid arthritis (RA) is a systemic autoimmune disease characterized by chronic destructive inflammation in synovial joints. To date, many studies explored the associations between tumor necrosis factor alpha inducible protein 3 (TNFAIP3) gene rs6920220, rs2230926, and rs5029937 polymorphisms and the risk of rheumatoid arthritis (RA), but with contradictory results. We therefore conducted a comprehensive meta-analysis to address the associations. We searched in the databases of PubMed and Embase. Pooled odds ratios (ORs) and 95% confidence intervals (CIs) were calculated by the Stata 11.0 software. A total of 21 case-control studies for these three single nucleotide polymorphisms (SNPs) were included in this meta-analysis. Meta-analysis indicated that TNFAIP3 gene rs6920220, rs2230926, and rs5029937 polymorphisms were associated with the increased risk of RA. Stratification analysis of ethnicity found that rs6920220 and rs5029937 polymorphisms increased the risk of RA among Caucasians, while rs2230926 polymorphism increased the risk of RA among Asians. In summary, this meta-analysis confirms that TNFAIP3 gene polymorphisms may play important roles in the pathogenesis of RA.

## INTRODUCTION

Rheumatoid arthritis (RA) is an autoimmune inflammatory disease, which is characterized by inflammation and destruction of synovial joints leading to progressive joint damage and disability. The etiology of RA is still poorly understood. RA is a multifactorial disorder, involving both genetic and environmental risk factors [1]. Studies indicate that genetic factors may be account for approximately 50–65% of the risk of RA [2]. Human leukocyte antigen (HLA) alleles are well recognized to be implicated in the pathogenesis of RA [3]. Many genome-wide association studies (GWASs) have confirmed known and identified new genetic determinants of RA [4].

The tumor necrosis factor alpha inducible protein 3 (TNFAIP3) gene encodes ubiquitin-editing protein A20 [5]. A20 is a potent anti-inflammatory protein, which is required for the termination of both tumor necrosis factor (TNF) and Toll-like receptor-induced NF-kB signals [5, 6]. The ubiquitin modifying enzyme A20 restricts B cell survival and prevents autoimmunity [7].TNFAIP3 could deregulate NF-κB-dependent gene expression via deubiquitinating specific NF-κB signaling molecules [6]. TNFAIP3 gene is located at 6q23, and is reported to be significantly associated with autoimmune diseases, including RA [8]. Recently, a host of studies [9–29] explored the associations between TNFAIP3 gene rs6920220, rs2230926, and rs5029937 polymorphisms and RA risk, but with contradictory results. These studies were conflicting and inconclusive due to clinical heterogeneity, different ethnic populations, and small sample sizes. In order to provide a convincing relationship between TNFAIP3 gene rs6920220, rs2230926, and rs5029937 polymorphisms and RA susceptibility, we performed this comprehensive meta-analysis to clarify the possible associations.

## RESULTS

### Characteristics of the included studies

**Figure 1:**
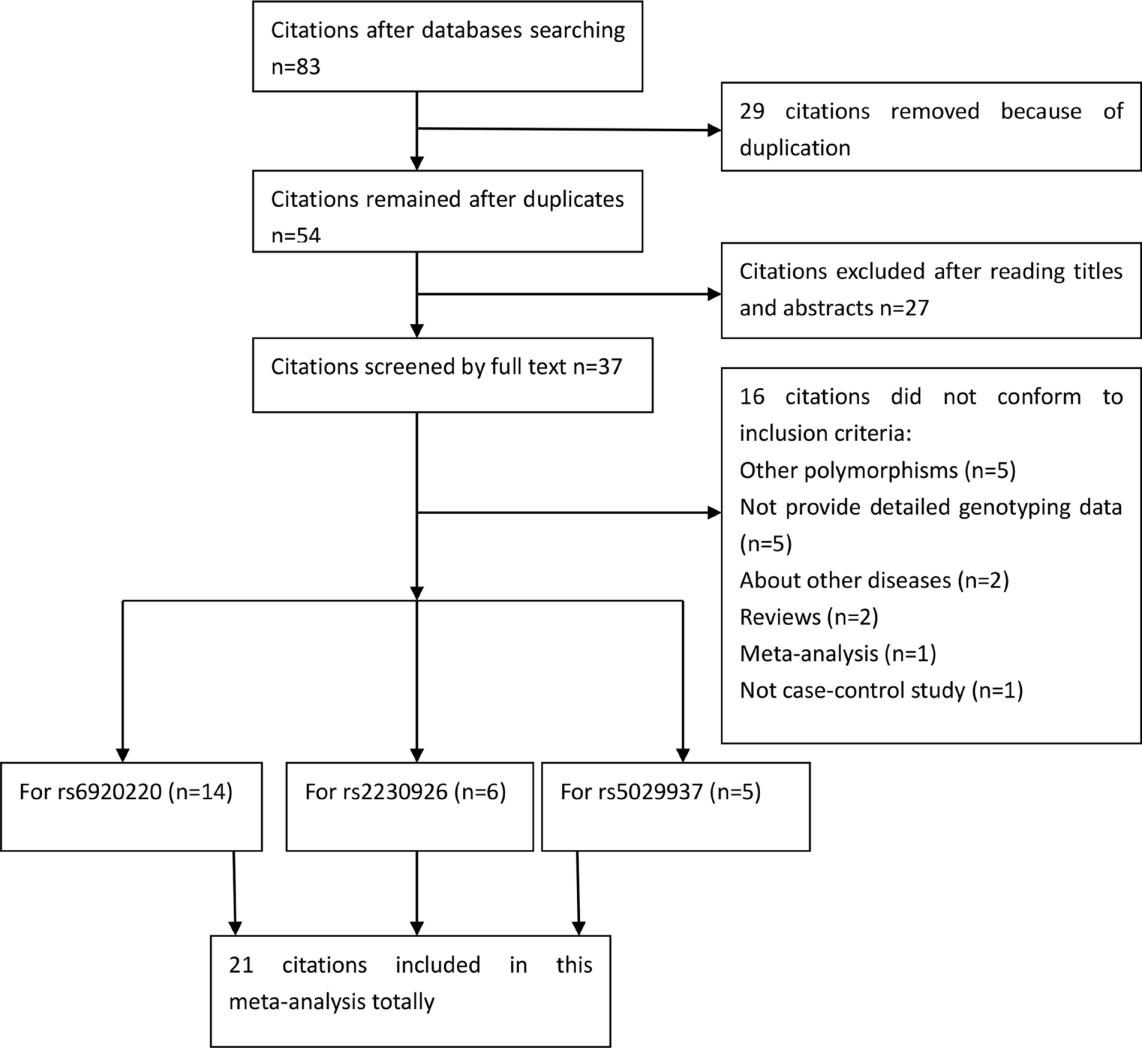
Selection for eligible publications included in this meta-analysis

Selection for eligible studies included in this meta-analysis was presented in Figure 1. We yielded a total of 83 citations after initial search. 29 citations were removed after removing duplicates. After screening the titles and abstracts, 27 citations were deleted. 37 citations were selected for further full text review. 16 citations were excluded because they did not conform to the inclusion criteria (see Figure 1). We finally identified 21 studies (27,451 cases and 30,443 controls) in this meta-analysis. 14 studies [9–13, 18, 20, 21, 23, 26–29] with 21,040 cases and 23,086 controls examined rs6920220 polymorphism; 6 studies [14, 17, 19, 22, 24, 25] including 5,912 cases and 6,463 controls investigated rs2230926 polymorphism; 5 studies [16, 18, 21, 23, 24] involving 12,518 cases and 14,061 controls explored rs5029937 polymorphism. The characteristics of included studies were summarized in Table 1. The Newcastle-Ottawa Scale (NOS) scores of all included studies ranged from 5 to 7 stars, suggesting that these studies were of high methodological quality.

**Table 1:** Characteristics of included studies

Author and year	Country	Genotype methods	Ethnicity	Case			Control			HWE	NOS
rs6920220				GG	GA	AA	GG	GA	AA		
Hegab2016	Egypt	TaqMan	Caucasian	378	15	1	382	15	1	0.0497	6
Maxwell2012	UK	Unclear	Caucasian	132	123	16	170	86	8	0.465	6
Ben Hamad2012	Tunisia	TaqMan	Tunisian	77	56	8	116	65	10	0.820	7
Hughes2010	USA	PCR	African-American	450	100	6	626	155	10	0.908	6
Morgan2010	UK	PCR	Caucasian	464	303	43	2146	1197	135	0.045	5
Plant2010	Mixed	PCR	Caucasian	1963	1044	139	2538	1349	179	0.988	7
Han2009	Korea	Unclear	Asian	1307	5	0	962	13	0	0.834	6
Stark2009	Slovak	PCR	Caucasian	324	175	16	213	78	7	0.964	6
Orozco2009	UK	PCR	Caucasian	2173	1449	221	2143	1188	136	0.070	7
Dieguez-Gonzalez2009	Spain	PCR	Caucasian	1004	567	80	1034	520	65	0.970	6
Perdigones2009	Spain	TaqMan	Caucasian	391	204	30	421	197	24	0.873	6
Lee2009	Korea	PCR	Asian	1110	3	0	986	1	0	0.987	6
Thomson2007	UK	PCR	Caucasian	2713	1816	277	2287	1266	142	0.041	6
Burton2007	UK	TaqMan	Caucasian	1007	723	127	1757	1049	129	0.078	6
rs2230926				TT	TG	GG	TT	TG	TT		
Hao2014	China	TaqMan	Asian	170	34	3	184	13	2	0.005	7
Zhang2014	China	TaqMan	Asian	1072	200	8	1133	143	4	0.819	5
Kim2014	Korea	Unclear	Asian	364	52	0	367	45	0	0.241	6
Perkins2012	USA	TaqMan	African American	177	208	61	282	345	106	0.977	7
Musone2011	USA	Unclear	Caucasian	133	14	1	1430	82	1	0.874	6
Shimane2010	Japan	Unclear	Asian	2815	571	29	2016	299	11	0.981	6
rs5029937				GG	GT	TT	GG	GT	TT		
Vernerova2016	Slovakia	TaqMan	Caucasian	477	21	1	850	43	1	0.554	6
Kim2014	Korea	Unclear	Asian	364	54	1	379	43	0	0.270	7
Maxwell2012	UK	Unclear	Caucasian	227	29	0	236	18	0	0.558	5
Plant2010	Mixed	PCR	Caucasian	6977	735	19	8847	547	9	0.856	6
Orozco2009	UK	PCR	Caucasian	3291	309	13	2876	207	5	0.528	6

HWE, Hardy-Weinberg equilibrium; NOS, Newcastle-Ottawa Scale

### Associations of the TNFAIP3 gene polymorphisms with RA susceptibility

**Figure 2:**
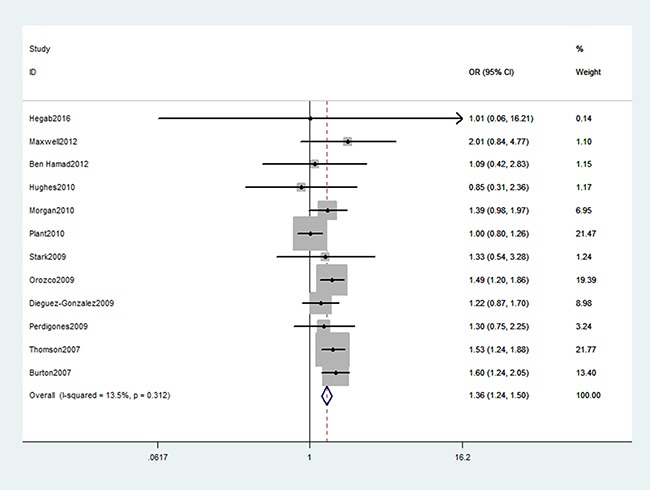
Forest plot shows odds ratio for the associations between rs6920220 polymorphism and RA risk (AA vs. GA+GG)

**Figure 3:**
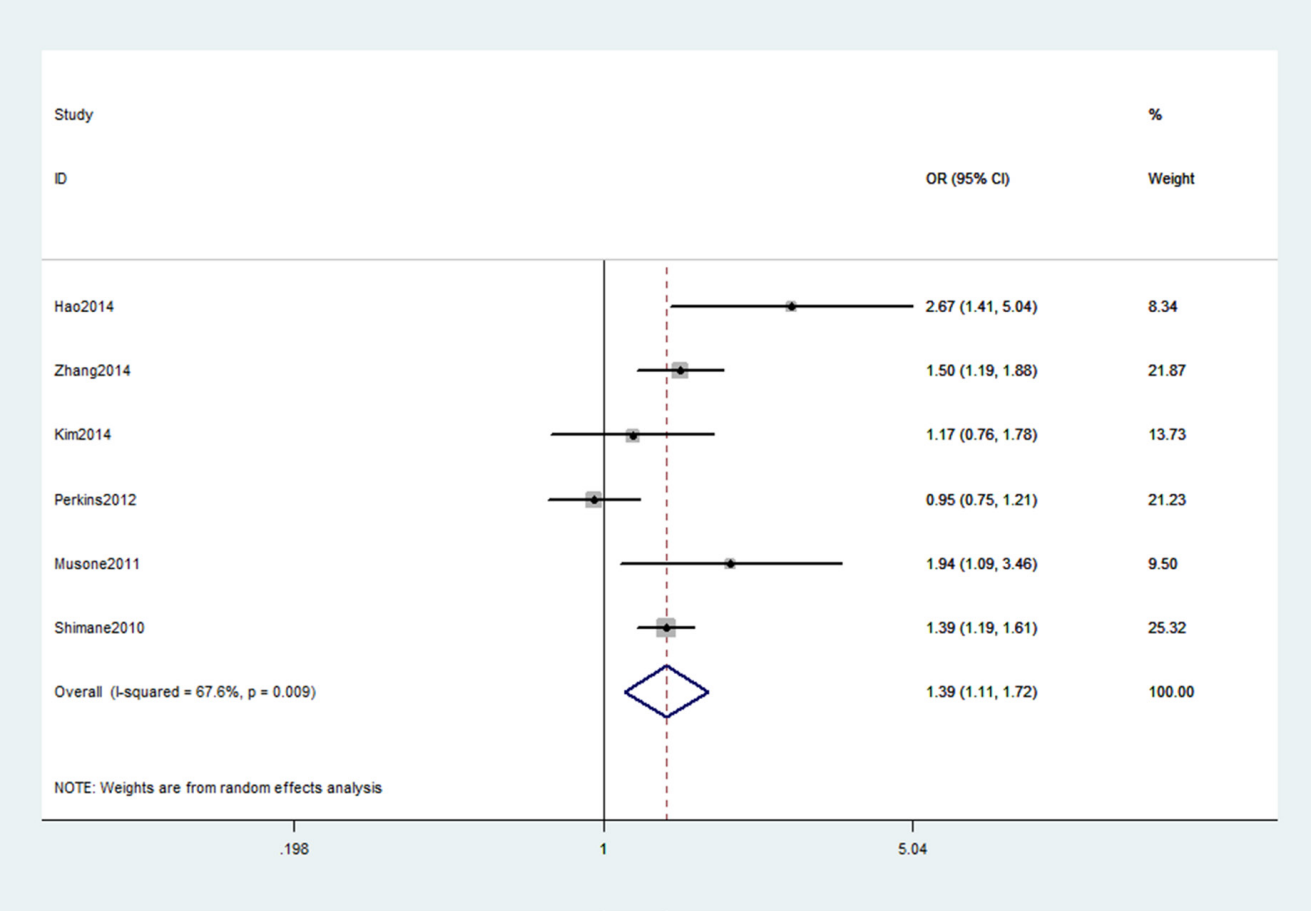
Forest plot shows odds ratio for the associations between rs2230926 polymorphism and RA risk (TG+GG vs. TT)

**Figure 4:**
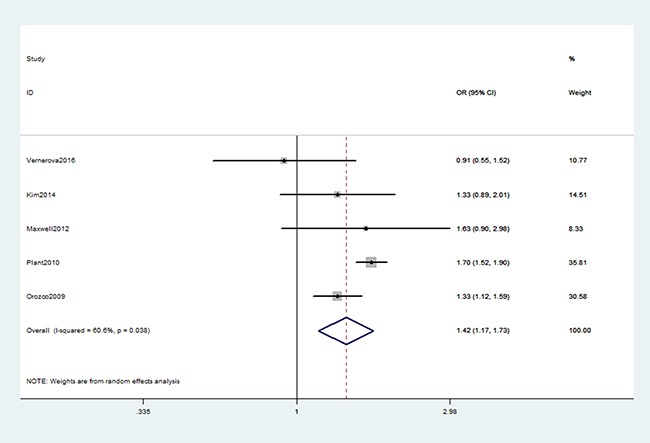
Forest plot shows odds ratio for the associations between rs5029937 polymorphism and RA risk (T vs. G)

As shown in Table 2, we found a significant association between TNFAIP3 gene rs6920220 (AA vs. GA+GG: OR, 1.36; 95% CI, 1.24–1.50, *P* < 0.001, Figure 2), rs2230926 (TG+GG vs. TT: OR, 1.39; 95% CI, 1.11–1.72, *P* = 0.003, Figure 3), and rs5029937 (T vs. G: OR, 1.42; 95% CI, 1.17–1.73, *P* < 0.001, Figure 4) polymorphisms with the increased risk of RA. Stratification analysis of ethnicity indicated that rs6920220 and rs5029937 polymorphisms increased the risk of RA among Caucasians, while rs2230926 polymorphism among increased the risk of RA among Asians. Similar results were obtained among all included studies when conducted stratification analysis of HWE status (Table 3).

**Table 2:** Meta-analysis of association between TNFAIP3 rs6920220, rs5029937 and rs2230926 polymorphisms and RA risk

Comparison	OR(95%CI)	*P*-value	P for heterogeneity	I^2^ (%)	Model
**rs6920220**					
A vs. G	**1.17(1.08,1.26)**	< 0.001	< 0.001	67.3	Random
GA+AA vs. GG	**1.19(1.09,1.29)**	< 0.001	< 0.001	65.2	Random
AA vs. GA+GG	**1.36(1.24,1.50)**	< 0.001	0.312	13.5	Fixed
**rs5029937**					
T vs. G	**1.42(1.17,1.73)**	< 0.001	0.038	60.6	Random
GT+TT vs. GG	**1.42(1.15,1.75)**	0.001	0.028	63.2	Random
TT vs. GT+GG	**2.40(1.30,4.44)**	0.005	0.955	0.0	Fixed
**rs2230926**					
G vs. T	**1.37(1.10,1.71)**	0.005	0.001	75.7	Random
TG+GG vs. TT	**1.39(1.11,1.72)**	0.003	0.009	67.6	Random
GG vs. TT+TG	1.14(0.86,1.52)	0.358	0.180	36.2	Fixed

^*^Bold values are statistically significant (P < 0.05).

**Table 3:** Summary of the subgroup analyses in this meta-analysis

Comparison	Category	Category	Studies	OR (95% CI)	*P*-value
**rs6920220**					
A vs. G	Ethnicity	Caucasian	10	**1.19(1.11,1.28)**	< 0.001
		Tunisian	1	1.20(0.84,1.72)	0.326
		African–American	1	0.90(0.70,1.16)	0.412
		Asian	2	0.69(0.08,5.89)	0.733
	HWE status	negative	3	**1.22(1.14,1.30)**	< 0.001
		positive	11	**1.16(1.05,1.28)**	0.004
GA+AA vs. GG	Ethnicity	Caucasian	10	**1.21(1.12,1.31)**	< 0.001
		Tunisian	1	1.29(0.83,2.00)	0.264
		African–American	1	0.89(0.68,1.17)	0.419
		Asian	2	0.69(0.08,5.92)	0.733
	HWE status	negative	3	**1.24(1.15,1.33)**	< 0.001
		positive	11	**1.18(1.05,1.32)**	0.005
AA vs. GA+GG	Ethnicity	Caucasian	10	**1.37(1.24,1.51)**	< 0.001
		Tunisian	1	1.09(0.42,2.83)	0.862
		African–American	1	0.85(0.31,2.36)	0.758
	HWE status	negative	3	**1.49(1.25,1.78)**	< 0.001
		positive	9	**1.31(1.17,1.47)**	< 0.001
**rs2230926**					
G vs. T	Ethnicity	Asian	4	**1.42(1.21,1.67)**	< 0.001
		African–American	1	0.96(0.81,1.14)	0.627
		Caucasian	1	**2.00(1.16,3.46)**	0.013
	HWE status	negative	1	**2.40(1.34,4.30)**	0.003
		positive	5	**1.29(1.04,1.60)**	0.020
TG+GG vs. TT	Ethnicity	Asian	4	**1.45(1.21,1.74)**	< 0.001
		African–American	1	0.95(0.75,1.21)	0.678
		Caucasian	1	**1.94(1.09,3.46)**	0.024
	HWE status	negative	1	**2.67(1.41,5.04)**	0.002
		positive	5	**1.30(1.07,1.59)**	0.010
GG vs. TT+TG	Ethnicity	Asian	3	**1.81(1.02,3.20)**	0.042
		African–American	1	0.94(0.67,1.32)	0.708
		Caucasian	1	10.29(0.64,165.29)	0.100
	HWE status	negative	1	1.45(0.24,8.76)	0.687
		positive	4	1.47(0.80, 2.70)	0.220
**rs5029937**					
T vs. G	Ethnicity	Caucasian	4	**1.43(1.14,1.79)**	0.002
		Asian	1	1.33(0.89,2.01)	0.168
GT+TT vs. GG	Ethnicity	Caucasian	4	**1.43(1.12,1.82)**	0.004
		Asian	1	1.33(0.87,2.04)	0.185

^*^Bold values are statistically significant (*P* < 0.05)

**Figure 5:**
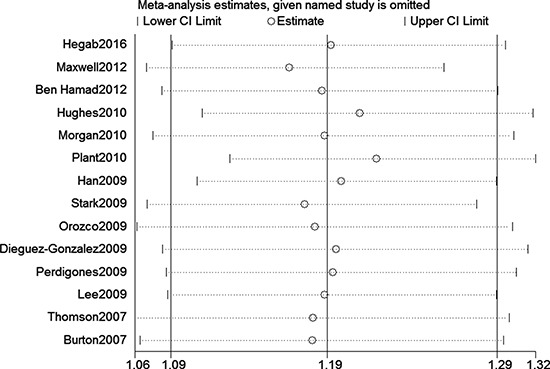
Sensitivity analyses for the associations between rs6920220 polymorphism and RA risk (GA+AA vs. GG)

**Figure 6:**
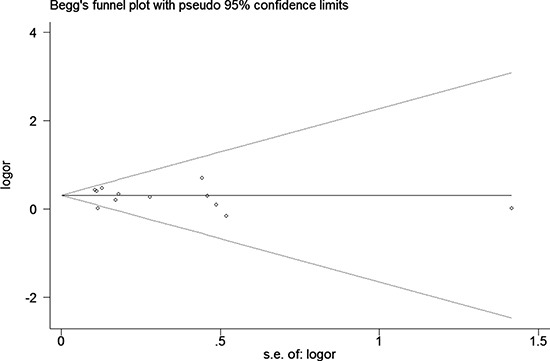
Begg's tests between rs6920220 polymorphism and RA risk (AA vs. GA+GG)

We assessed sensitivity by omitting each study once at a time in every genetic model for the three polymorphisms. This meta-analysis indicated that the data of these three single nucleotide polymorphisms (SNPs) (rs6920220, GA+AA vs. GG, Figure 5) were stable and trustworthy. Both Egger's and Begg's tests (rs6920220, AA vs. GA+GG, Figure 6) were used to evaluated the publication bias of this meta-analysis. Our data revealed that there was no obvious publication bias for above polymorphisms (data not shown).

## DISCUSSION

In this meta-analysis, our data found that TNFAIP3 gene rs6920220, rs2230926, and rs5029937 polymorphisms increased the risk of RA. Stratification analysis of ethnicity indicated that rs6920220 and rs5029937 polymorphisms increased the risk of RA among Caucasians, while rs2230926 polymorphism among increased the risk of RA among Asians.

TNFAIP3 is an inhibitor of the NF-κB signaling pathway, which is significantly associated with the development of RA [30]. Vereecke et al. illustrated the importance of TNFAIP3 in the resolution of inflammation and the prevention of RA [31]. TNFAIP3 gene involves in the negative regulation of inflammatory responses, and alters the expression or activity of A20, which influence the pathogenesis of RA [7]. A meta-analysis performed by Lee et al. investigated TNFAIP3 gene rs6920220, rs2230926 polymorphisms with RA susceptibility recently [32]. They found that rs6920220 and rs2230926 polymorphisms were associated with the increased risk of RA, which is consistent with our results. The findings of this present meta-analysis regarding the association between rs6920220, rs2230926 polymorphisms and RA in Caucasians and Asians are mostly in agreement with the previous meta-analysis by Lee et al.y [32]. However, our data showed no association of rs2230926 polymorphism with RA in African–Americans, unlike the positive result from Lee et al. [32]. Furthermore, our data about rs2230926 polymorphism among African–Americans was also in accordance with findings of original study by Perkins et al. from America [19], indicating that the data of Lee et al. was not trustworthy. We also found rs2230926 polymorphism was associated with the risk of RA among Caucasians, which was not uncovered by previous meta-analysis [32]. Another notable limitation of the meta-analysis by Lee et al. was that they did not include several studies of rs6920220 polymorphism [11, 15, 18, 20, 21, 28], which actually met the inclusion criteria of their meta-analysis. Therefore, we assumed previous meta-analysis could not provide a comprehensive conclusion. Furthermore, Additional studies [9, 14, 23–25] have been published in recent years since the meta-analysis. The findings of these studies were conflicting. Distribution of gene functional polymorphisms varying in different races, inadequate statistical power of single study, clinical heterogeneity, small sample size, or uncorrected multiple hypothesis testing may contribute to the inconsistent findings. In order to overcome these limitations, it is necessary to conduct a new meta-analysis including the updated data to confirm whether the TNFAIP3 gene polymorphisms are associated with RA susceptibility.

We believe our meta-analysis has some strengths over previous meta-analysis Lee et al. for the following reasons. First, we included 14 studies with 21,040 cases and 23,086 controls examining rs6920220 polymorphism and 6 studies with 5,912 cases and 6,463 controls investigating rs2230926 polymorphism, indicating that the sample sizes of rs6920220 and rs2230926 polymorphisms were large. Second, we conducted sensitivity analysis and power analysis, suggesting that our data about these SNPs were trustworthy and robust. Third, we also conducted a meta-analysis of another SNP of TNFAIP3 gene (rs5029937 polymorphism). The data revealed that rs5029937 polymorphism increased the risk of RA. Stratification analysis of ethnicity also found a positive association between rs5029937 polymorphism and RA among Caucasians, but not Asians. There are several possible interpretations for different results of these SNPs between Asians and Caucasians. First, genetic heterogeneity for RA may exist in different populations. Second, the discrepancy may be explained by clinical heterogeneity between the different populations. Third, the sample sizes of the Asian populations might not have been sufficiently large to reach a convincing conclusion when compared with Caucasian populations. Additionally, the different genotyping methods and random errors may also been potential reasons for different findings between Asians and Caucasians. To our knowledge, this is the first meta-analysis to address the association between rs5029937 polymorphism and RA risk.

Several potential limitations should be addressed in this meta-analysis. First, due to limited data, we could not perform further stratification analyses of other potential factors, such as rheumatoid factor (RF). Second, our results were based on unadjusted estimates for confounding factors, which might have affected the final conclusions. Third, we could not assess potential gene-gene and gene-environment interactions due to the lack of relevant data. Fourth, we cannot examine the associations between these SNPs of TNFAIP3 and the clinical manifestations of RA. Fifth, some genetic models of this meta-analysis were high, and it is necessary to interpret it with caution. Sixth, the sample sizes of stratification analyses were limited.

In conclusion, this meta-analysis indicates that TNFAIP3 gene rs6920220, rs2230926, and rs5029937 polymorphisms are associated with the increased risk of RA. Stratification analysis of ethnicity reveals that rs6920220 and rs5029937 polymorphisms increase the risk of RA among Caucasians, while rs2230926 polymorphism increases the risk of RA among Asians. Further studies are required to determine whether these SNPs of TNFAIP3 gene contribute to RA susceptibility in different ethnic groups.

## MATERIALS AND METHODS

### Literature search

We systematically searched the PubMed and Embase to identify studies through September 16, 2016. The following search terms were used: “tumor necrosis factor alpha inducible protein 3,” ‘‘TNFAIP3,’’ ‘‘A20,’’ ‘‘Rheumatoid Arthritis,’’ ‘‘RA,’’ ‘‘polymorphism,’’ ‘‘SNP’’ and ‘‘polymorphisms’’. No restrictions were placed on the search. Additional initially omitted studies (such as reference lists of identified studies) have been identified by hand screening. The identified studies conformed to the following criteria: studies that evaluated the association between RA risk and TNFAIP3 gene rs6920220, rs2230926, and rs5029937 polymorphisms, study provided sufficient data to calculate the odds ratios (ORs) and 95% confidence intervals (CIs), and *P* value, and case-control study. Exclusion criteria were: duplication of previous studies; review, or other non-original studies; studies without detailed genotype data.

### Data extraction and quality assessment

Relevant information was carefully extracted from all eligible studies. The extracted information from all eligible studies included: name of first author, publication year, country of origin, genotype methods, ethnicity, and genotype numbers of cases and controls. Two reviewers independently performed the extraction of data and assessed the study quality based on the NOS [33]. All disagreements were discussed and resolved with consensus.

### Statistical analysis

All statistical analyses were performed using the Stata 11.0 software (StataCorp, College Station, TX, USA). ORs and 95%CIs were used to assess the strength of associations between TNFAIP3 gene rs6920220, rs2230926, and rs5029937 polymorphisms and RA risk. Stratification analyses were carried out by ethnicity and HWE status. When a Q test indicated *P* < 0.1 or I^2^ > 50% indicated heterogeneity across studies, a random-effect model was used. Otherwise, the fixed-effects model was applied [34]. Pooled ORs were calculated for allele model, dominant model, and recessive model. We performed sensitivity analyses by omitting each study in turn to determine the effect on the test of heterogeneity and evaluated the stability of the overall results. We assessed the departure from the HWE in the controls using Pearson's χ^2^ test. Potential publication bias was assessed by Begger's and Egger's linear regression test [35]; *P* < 0.05 was considered to indicate statistically significant. The power of this meta-analysis was calculated with a significant value of 0.05 [36].

## Abbreviations

RA: rheumatoid arthritis
RF: rheumatoid factor
TNFAIP3: tumor necrosis factor alpha inducible protein 3
GWAS: genome-wide association study
HLA: Human leukocyte antigen
HWE: Hardy–Weinberg equilibrium
CI: confidence interval
OR: odds ratio
NOS: Newcastle-Ottawa Scale
SNP: single nucleotide polymorphism

## References

[1] Smolen JS, Aletaha D, McInnes IB (2016). Rheumatoid arthritis. Lancet.

[2] MacGregor AJ, Snieder H, Rigby AS, Koskenvuo M, Kaprio J, Aho K, Silman AJ (2000). Characterizing the quantitative genetic contribution to rheumatoid arthritis using data from twins. Arthritis Rheum.

[3] Kurko J, Besenyei T, Laki J, Glant TT, Mikecz K, Szekanecz Z (2013). Genetics of rheumatoid arthritis - a comprehensive review. Clin Rev Allergy Immunol.

[4] Bowes J, Barton A (2008). Recent advances in the genetics of RA susceptibility. Rheumatology (Oxford).

[5] Boone DL, Turer EE, Lee EG, Ahmad RC, Wheeler MT, Tsui C, Hurley P, Chien M, Chai S, Hitotsumatsu O, McNally E, Pickart C, Ma A (2004). The ubiquitin-modifying enzyme A20 is required for termination of Toll-like receptor responses. Nat Immunol.

[6] Wertz IE, O’Rourke KM, Zhou H, Eby M, Aravind L, Seshagiri S, Wu P, Wiesmann C, Baker R, Boone DL, Ma A, Koonin EV, Dixit VM (2004). De-ubiquitination and ubiquitin ligase domains of A20 downregulate NF-kappaB signalling. Nature.

[7] Tavares RM, Turer EE, Liu CL, Advincula R, Scapini P, Rhee L, Barrera J, Lowell CA, Utz PJ, Malynn BA, Ma A (2010). The ubiquitin modifying enzyme A20 restricts B cell survival and prevents autoimmunity. Immunity.

[8] Hamerman JA, Pottle J, Ni M, He Y, Zhang ZY, Buckner JH (2016). Negative regulation of TLR signaling in myeloid cells--implications for autoimmune diseases. Immunol Rev.

[9] Hughes LB, Reynolds RJ, Brown EE, Kelley JM, Thomson B, Conn DL, Jonas BL, Westfall AO, Padilla MA, Callahan LF, Smith EA, Brasington RD, Edberg JC (2010). Most common single-nucleotide polymorphisms associated with rheumatoid arthritis in persons of European ancestry confer risk of rheumatoid arthritis in African Americans. Arthritis Rheum.

[10] Thomson W, Barton A, Ke X, Eyre S, Hinks A, Bowes J, Donn R, Symmons D, Hider S, Bruce IN, Wilson AG, Marinou I, Wellcome Trust Case Control C (2007). Rheumatoid arthritis association at 6q23. Nature genetics.

[11] Morgan AW, Robinson JI, Conaghan PG, Martin SG, Hensor EM, Morgan MD, Steiner L, Erlich HA, Gooi HC, Barton A, Worthington J, Emery P, Consortium U (2010). Evaluation of the rheumatoid arthritis susceptibility loci HLA-DRB1, PTPN22, OLIG3/TNFAIP3, STAT4 and TRAF1/C5 in an inception cohort. Arthritis Res Ther.

[12] Hegab MM, Abdelwahab AF, El-Sayed Yousef AM, Salem MN, El-Baz W, Abdelrhman S, Elshabacy F, Alhefny A, Abouraya W, Ibrahim SM, Ragab G (2016). CD28 and PTPN22 are associated with susceptibility to rheumatoid arthritis in Egyptians. Hum Immunol.

[13] Dieguez-Gonzalez R, Calaza M, Perez-Pampin E, Balsa A, Blanco FJ, Canete JD, Caliz R, Carreno L, AR de la Serna, Fernandez-Gutierrez B, Ortiz AM, Herrero-Beaumont G, Pablos JL (2009). Analysis of TNFAIP3, a feedback inhibitor of nuclear factor-kappaB and the neighbor intergenic 6q23 region in rheumatoid arthritis susceptibility. Arthritis Res Ther.

[14] Zhang X, Li W, Zhang X, Zhao L, Zhang X, Jiang L, Guo Y, Zhang J, Liang Z, Wang X (2014). Single nucleotide polymorphisms in TNFAIP3 were associated with the risks of rheumatoid arthritis in northern Chinese Han population. BMC Med Genet.

[15] Wellcome Trust Case Control C (2007). Genome-wide association study of 14,000 cases of seven common diseases and 3,000 shared controls. Nature.

[16] Vernerova L, Spoutil F, Vlcek M, Krskova K, Penesova A, Meskova M, Marko A, Raslova K, Vohnout B, Rovensky J, Killinger Z, Jochmanova I, Lazurova I (2016). A Combination of CD28 (rs1980422) and IRF5 (rs10488631) Polymorphisms Is Associated with Seropositivity in Rheumatoid Arthritis: A Case Control Study. PloS one.

[17] Shimane K, Kochi Y, Horita T, Ikari K, Amano H, Hirakata M, Okamoto A, Yamada R, Myouzen K, Suzuki A, Kubo M, Atsumi T, Koike T (2010). The association of a nonsynonymous single-nucleotide polymorphism in TNFAIP3 with systemic lupus erythematosus and rheumatoid arthritis in the Japanese population. Arthritis Rheum.

[18] Plant D, Flynn E, Mbarek H, Dieude P, Cornelis F, Arlestig L, Dahlqvist SR, Goulielmos G, Boumpas DT, Sidiropoulos P, Johansen JS, Ornbjerg LM, Hetland ML (2010). Investigation of potential non-HLA rheumatoid arthritis susceptibility loci in a European cohort increases the evidence for nine markers. Ann Rheum Dis.

[19] Perkins EA, Landis D, Causey ZL, Edberg Y, Reynolds RJ, Hughes LB, Gregersen PK, Kimberly RP, Edberg JC, Bridges SL (2012). and Consortium for the Longitudinal Evaluation of African Americans with Early Rheumatoid Arthritis I. Association of single-nucleotide polymorphisms in CCR6, TAGAP, and TNFAIP3 with rheumatoid arthritis in African Americans. Arthritis Rheum.

[20] Perdigones N, Lamas JR, Vigo AG, de la Concha EG, Jover JA, Urcelay E, Gutierrez BF, Martinez A (2009). 6q23 polymorphisms in rheumatoid arthritis Spanish patients. Rheumatology (Oxford).

[21] Orozco G, Hinks A, Eyre S, Ke X, Gibbons LJ, Bowes J, Flynn E, Martin P, consortium Y, Wilson AG, Bax DE, Morgan AW, Wellcome Trust Case Control C (2009). Combined effects of three independent SNPs greatly increase the risk estimate for RA at 6q23. Human molecular genetics.

[22] Musone SL, Taylor KE, Nititham J, Chu C, Poon A, Liao W, Lam ET, Ma A, Kwok PY, Criswell LA (2011). Sequencing of TNFAIP3 and association of variants with multiple autoimmune diseases. Genes Immun.

[23] Maxwell JR, Gowers IR, Kuet KP, Barton A, Worthington J, Wilson AG (2012). Expression of the autoimmunity associated TNFAIP3 is increased in rheumatoid arthritis but does not differ according to genotype at 6q23. Rheumatology (Oxford).

[24] Kim SK, Choe JY, Bae J, Chae SC, Park DJ, Kwak SG, Lee SS (2014). TNFAIP3 gene polymorphisms associated with differential susceptibility to rheumatoid arthritis and systemic lupus erythematosus in the Korean population. Rheumatology (Oxford).

[25] Hao G, Li Y, Liu J, Wo M (2014). TNFAIP3 rs2230926 polymorphisms in rheumatoid arthritis of southern Chinese Han population: a case-control study. Int J Clin Exp Pathol.

[26] Han TU, Bang SY, Kang C, Bae SC (2009). TRAF1 polymorphisms associated with rheumatoid arthritis susceptibility in Asians and in Caucasians. Arthritis Rheum.

[27] Stark K, Rovensky J, Blazickova S, Grosse-Wilde H, Ferencik S, Hengstenberg C, Straub RH (2009). Association of common polymorphisms in known susceptibility genes with rheumatoid arthritis in a Slovak population using osteoarthritis patients as controls. Arthritis Res Ther.

[28] Lee HS, Korman BD, Le JM, Kastner DL, Remmers EF, Gregersen PK, Bae SC (2009). Genetic risk factors for rheumatoid arthritis differ in Caucasian and Korean populations. Arthritis Rheum.

[29] Ben Hamad M, Cornelis F, Maalej A, Petit-Teixeira E (2012). A Tunisian case-control association study of a 6q polymorphism in rheumatoid arthritis. Rheumatology international.

[30] Lee EG, Boone DL, Chai S, Libby SL, Chien M, Lodolce JP, Ma A (2000). Failure to regulate TNF-induced NF-kappaB and cell death responses in A20-deficient mice. Science.

[31] Vereecke L, Beyaert R, van Loo G (2009). The ubiquitin-editing enzyme A20 (TNFAIP3) is a central regulator of immunopathology. Trends Immunol.

[32] Lee YH, Bae SC, Choi SJ, Ji JD, Song GG (2012). Associations between TNFAIP3 gene polymorphisms and rheumatoid arthritis: a meta-analysis. Inflamm Res.

[33] Stang A (2010). Critical evaluation of the Newcastle-Ottawa scale for the assessment of the quality of nonrandomized studies in meta-analyses. Eur J Epidemiol.

[34] Higgins JP, Thompson SG (2002). Quantifying heterogeneity in a meta-analysis. Stat Med.

[35] Peters JL, Sutton AJ, Jones DR, Abrams KR, Rushton L (2006). Comparison of two methods to detect publication bias in meta-analysis. Jama.

[36] Hedges LV, Pigott TD (2001). The power of statistical tests in meta-analysis. Psychol Methods.

